# Post-Traumatic Stress Disorder in Chinese Teachers during COVID-19 Pandemic: Roles of Fear of COVID-19, Nomophobia, and Psychological Distress

**DOI:** 10.3390/healthcare9101288

**Published:** 2021-09-28

**Authors:** Shikha Kukreti, Daniel Kwasi Ahorsu, Carol Strong, I-Hua Chen, Chung-Ying Lin, Nai-Ying Ko, Mark D. Griffiths, Yu-Pin Chen, Yi-Jie Kuo, Amir H. Pakpour

**Affiliations:** 1 Department of Public Health, National Cheng Kung University Hospital, College of Medicine, National Cheng Kung University, Tainan 701, Taiwan; t88087025@gs.ncku.edu.tw (S.K.); carolcj@ncku.edu.tw (C.S.); 2 Department of Rehabilitation Sciences, Faculty of Health & Social Sciences, The Hong Kong Polytechnic University, Hung Hom, Hong Kong; daniel.ahorsu@connect.polyu.hk; 3 Chinese Academy of Education Big Data, Qufu Normal University, Qufu 273165, China; 4 Institute of Allied Health Sciences, College of Medicine, National Cheng Kung University, Tainan 701, Taiwan; 5 Department of Occupational Therapy, College of Medicine, National Cheng Kung University, Tainan 701, Taiwan; 6 Department of Nursing, College of Medicine, National Cheng Kung University, Tainan 701, Taiwan; nyko@mail.ncku.edu.tw; 7 International Gaming Research Unit, Psychology Department, Nottingham Trent University, Nottingham NG1 4FQ, UK; mark.griffiths@ntu.ac.uk; 8 Department of Orthopedic Surgery, Wan Fang Hospital, Taipei Medical University, Taipei 110, Taiwan; 99231@w.tmu.edu.tw (Y.-P.C.); benkuo5@tmu.edu.tw (Y.-J.K.); 9 Department of Orthopedic Surgery, School of Medicine, College of Medicine, Taipei Medical University, Taipei 110, Taiwan; 10 Department of Nursing, School of Health and Welfare, Jönköping University, 55318 Jönköping, Sweden; apakpour@qums.ac.ir

**Keywords:** SARS-CoV-2, PTSD, fear, teacher

## Abstract

There are limited data concerning the prevalence of post-traumatic stress disorder (PTSD) among teachers. Therefore, the present study estimated the prevalence of PTSD among mainland Chinese teachers during the COVID-19 pandemic and to construct a model with mediation and moderation effects to explain the PTSD. Data collection was conducted in schools in the Jiangxi province between October and November 2020 among k-12 schoolteachers. An online survey, including five different psychometric scales, was used to collect data. All participants were assessed for PTSD using the Chinese version of the PTSD Checklist for DSM-5 (PCL-5). Hayes’ PROCESS Model 8 was used to examine the potential factors explaining a higher PTSD scores. A total of 2603 teachers from k-12 schools participated. With the cutoff score at 31, the prevalence of PTSD was 12.3% but decreased to 1.0% when the cutoff score was at 49. Nomophobia moderated the effects of Fear of COVID-19 Scale on PTSD. The findings suggest that fear of COVID-19 among teachers leads to PTSD via psychological distress, highlighting the moderating effect of nomophobia in this association. Based on the study’s findings, psychological interventions and educational training are needed to reduce fear among teachers at higher risk of developing PTSD.

## 1. Introduction

The novel coronavirus disease-2019 (COVID-19) outbreak which emerged in December 2019 in Wuhan (China) rapidly spread overseas, leading to it being declared a Public Health Emergency of International Concern (PHEIC) on 30 January 2020, by the World Health Organization (WHO) Emergency Committee [1]. As a consequence of rapidly increasing numbers of confirmed cases and deaths, individuals have been experiencing various psychological problems such as psychological distress (including depression, anxiety, and stress) [2,3], fear [4], death distress [5,6], and post-traumatic stress disorders (PTSD) [5]. These concerns arose in all prior infectious outbreaks (e.g., MERS ([middle east respiratory syndrome] and SARS [severe acute respiratory syndrome]) that took place years ago [7,8]. However, the COVID-19 pandemic has been viewed as a unique threat that has increased the panic, stress, anxiety, and the potential for depression due to inadequate knowledge of its transmission, lack of preparedness of the health system, and/or the absence of a treatment protocol or vaccination program [9,10].

Several cohorts, irrespective of their occupation (e.g., students, teachers, employees), have suffered psychological stress, experienced reduced autonomy, and felt job insecurity due to the COVID-19 pandemic [11,12,13]. Considering the pandemic, the educational sector was one of the first to suffer after the announcement of interim measures to mitigate the spread of COVID-19 [14]. Consequently, teachers have had to contend with the strains of the pandemic itself. More specifically, they have health concerns for themselves and others, changes brought on by working from home, threats to the current job and future career, new family and domestic responsibilities, and were often confined to their home [15]. A study conducted in Sweden has shown that teachers were more severely affected by keeping lower-secondary schools open during the lockdown, and measures to protect them could be considered [16]. Therefore, the negative effects of the outbreak appear to have deleterious consequences on teachers.

Post-traumatic stress disorder (PSTD) is a psychological disorder that can occur when individuals experience a traumatic incident such as natural calamities or infectious disease outbreaks such as SARS or COVID-19 [17,18]. Its essential feature is the characteristic symptoms resulting from exposure to a traumatic experience, a personal disastrous event, which involves death, injury, or threat to others’ physical integrity [19,20]. Prior studies have reported the prevalence of PTSD for different populations. For example, the prevalence of PTSD during the COVID-19 outbreak has been reported at 2.7% among university students [21], with significantly higher prevalence rates of 57.1% among healthcare workers [22]. To be effective as both caregivers and educators for students, teachers’ mental health conditions should be addressed. Although students and teachers may suffer from the same disaster, the literature lacks any data regarding PTSD among teachers during the COVID-19 pandemic.

Prior studies have shown that participants with high levels of psychological distress often develop PTSD symptoms [4,5]. This is due to fear of injury and death among individuals, particularly due to unexpected and unprepared events, which can create panic, fear, and tension [23]. Furthermore, this unprepared for situation may cause psychological distress among individuals, which would result in substantial psychological stress and aggravating PTSD symptoms. One psychological aspect of the COVID-19 pandemic is fear. Fear of contagion of inappropriate magnitude may result in PTSD due to psychological distress [24]. Therefore, fear of COVID-19 may be a predictor of PTSD. However, the mediating and moderating mechanisms underlying fear of COVID-19 and PTSD require further investigation. Therefore, the present study proposed a potential mediation–moderation model to explain PTSD. More specifically, fear of COVID-19 is postulated as the explaining factor to PTSD via psychological distress as the mediator and nomophobia as moderator.

The term ‘nomophobia’ refers to the fear of not having a mobile phone or mobile phone contact [25]. During the COVID-19 pandemic, the dependency on smartphones has markedly increased [26] as they serve as a key communication device and have other benefits such as accessing online classes, listening to music, and playing digital games, etc. All these activities have the potential to exacerbate the feelings of anxiety if individuals are unable to use their smartphones [27]. Prior studies have shown that individuals experience increased episodes of anxiety, trembling, perspiration, tachycardia, respiratory alterations, depression, panic, and fear when they are unable to access their smartphone [28]. Further, empirical research supported the idea, indicating that individuals with nomophobia suffer from stress when their smartphones are out of reach [29]. A prior study by Stefan Tams et al. pointed out that if control of phone withdrawal is low or uncertainty of event is high, nomophobia will lead to stress [30]. Therefore, nomophobia could exacerbate the effect of fear of COVID-19 on PTSD as they share similar symptoms.

Moreover, fear of COVID-19 may be elevated when receiving scary information concerning COVID-19 via social media on smartphones. Consequently, when a person has higher levels of nomophobia, they may frequently check information and/or social media on a smartphone and obtain COVID-19 information that makes them scared. This may enhance the effect of fear of COVID-19 on PTSD. In order to test the speculation asserted here, this present study (i) estimated the prevalence of PTSD among mainland Chinese teachers during the COVID-19 pandemic, and (ii) constructed a moderated mediation model to explain PTSD. More specifically, the model tested whether the association between fear of COVID-19 and PTSD is mediated by psychological distress and moderated by nomophobia; whether the association between fear of COVID-19 and psychological distress is moderated by nomophobia.

## 2. Materials and Methods

### 2.1. Participants and Procedure

The data collection period was between 26 October and November 2020 via the online survey platform *Sojump*. A non-probability sampling strategy was employed for data collection. The research team first sought help from the principals of k-12 schools in Jiangxi province. After that, the principals who accepted the invitation to participate provided the online survey link to the schoolteachers. In order to minimize the potential biases caused by a small number of schools (i.e., teachers in the same school may share similar demographic characteristics), the present study attempted to invite as many schools as possible. Finally, a total of 188 schools participated. However, school principals who accepted the present study’s invitation might have high levels of confidence in their teachers’ mental health. The online survey was voluntary and anonymous. Informed consent was provided by all the participants using the e-forms. The *Sojump* platform prompted participants to complete all items. Therefore, there were no missing data in the study.

The participants were recruited from Jiangxi as it is a province close to Wuhan, where the COVID-19 outbreak originated. After the lockdown in Wuhan, some Wuhan residents traveled to Jiangxi province, which led to Jiangxi being one of the first regions in the world to experience a large-scale COVID-19 outbreak. Moreover, Jiangxi province is located in inner China, and its educational infrastructure is less developed than in the coastal provinces due to its less developed local economy. Therefore, much less attention was paid to the teacher populations by the authorities. More specifically, the psychological resources and psychological counseling services during the outbreak of the pandemic (March to June 2020) were scarce in Jiangxi. Only after September, 2020 (i.e., when the outbreak was under control), did Jiangxi province introduce a public welfare hotline for psychological assistance, which teachers and students were encouraged to use.

This study was approved by the Institutional Review Board of the Jianxi Psychological Consultant Association. The sample size needed for the present study was calculated using the following equation: *d* = Z √P(1 − P)/n, where n = sample size needed; Z = Z statistic for a level of confidence (set at 1.96 in the present study); P = Expected proportion (set at 0.01 in the present study), and *d* = Precision (set at 0.004 in the present study). The calculation indicated that 2377 participants were needed.

### 2.2. Measures

The *Chinese PTSD Checklist for DSM-5* (PCL-5) has 20 items that assess the 20 DSM-5 symptoms of PTSD with good reliability and validity [31,32]. It uses a five-point scale from 0 (*not at all*) to 4 (*extremely*). The PCL-5 is easy to administer and assesses all symptom clusters of DSM-5 PTSD over the past month. The total score of the PCL-5 is calculated by adding the scores of each item [33]. The study used two cutoff scores of 31 and 49. A cutoff score of 31 was used based on the suggestion of research indicating that a lower cutoff score should be considered to increase sensitivity and a higher cutoff score should be considered when attempting to minimize false positives [34]. Moreover, based on the suggestion of study by Fung et al. (2019), the present study used another cutoff score of 49 [33]. Fung et al. reported that a cutoff score of 49 appears to yield an optimal balance between sensitivity and specificity among adult samples. The Cronbach’s α of the PCL-5 in the present study was excellent (0.97).

The seven-item *Chinese Fear of COVID-19 Scale* (FCV-19S) assesses individuals’ fear of COVID-19 [35]. Items are responded to on a five-point scale from 1 (*strongly disagree*) to 5 (*strongly agree*). A higher score of the FCV-19S represents a greater level of fear of COVID-19. An example item is *“I cannot sleep because I’m worrying about getting COVID-19”* [36]. Higher scores indicate greater fear of COVID-19. The Cronbach’s α of the FCV-19S in the present study was very good (0.89).

The 20-item *Chinese Nomophobia Questionnaire* (NMPQ) was developed to assess the dimensions of nomophobia [37]. An example item is *“I would feel uncomfortable without constant access to information through my smartphone”*. The NMPQ comprises four factors (Factor 1: losing connectedness; Factor 2: giving up convenience; Factor 3: not being able to communicate; Factor 4: not being able to access information). Items are responded to on seven-point scale from 1 (*strongly disagree*) to 7 (*strongly agree*) is applied to each NMPQ item. The responses are summed up to find a total score. Higher scores indicate greater severity of nomophobia [38]. The Cronbach’s α of the NMPQ in the present study was excellent (0.97).

The *Chinese Depression, Anxiety, Stress Scale* (DASS-21) is a self-report scale assessing negative affect comprising three seven-item subscales (depression, anxiety, and stress). Items are responded to on a four-point scale from 0 (*“does not apply to me at all”*) to 3 (*“applies to me very much or most of the time”*). Scores for each subscale are obtained by summing the responses of the all the items [39]. Higher scores indicate greater negative experience in the past week. The Cronbach’s α of the DASS-21 in the present study was excellent (0.97).

All the instruments were adopted from the previous studies and these scales have demonstrated good internal and test–retest reliability in a Chinese sample [33,35,38,39]. In addition to the validated psychometric scales, the participants provided information regarding their gender, teaching experience, and school type (i.e., public or private school).

### 2.3. Data Analysis

**Figure 1:**
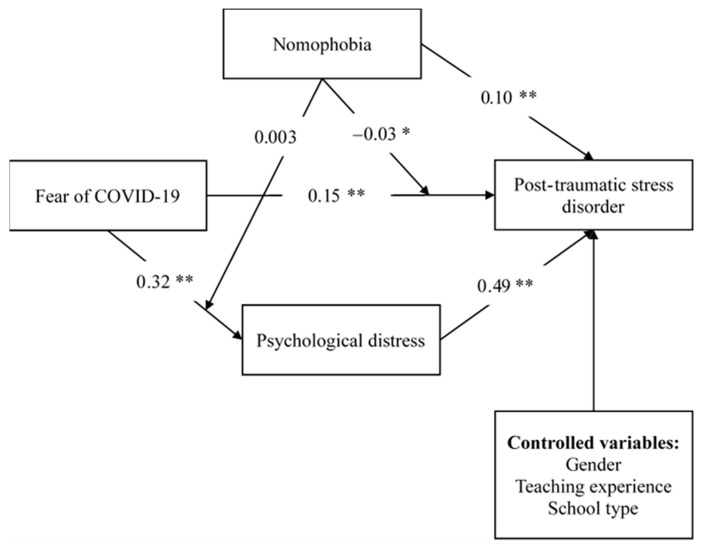
The moderated mediation model to explain post-traumatic stress disorder in Chinese teachers during COVID-19 pandemic. * *p* < 0.05; ** *p* < 0.00.

Descriptive statistics of means and frequencies were first applied to the data to understand the participants’ characteristics, including their background information and all the information assessed using the standardized scales. Pearson correlations were then carried out to examine the associations between every two studied variables. Finally, study used PROCESS Model 8 (i.e., moderated mediation model) suggested by Hayes [40] to examine potential reasons for higher PTSD scores. More specifically, FCV-19S score (assessing fear of COVID-19) was treated as the independent variable, NMPQ score (assessing nomophobia) as the moderated variable, DASS-21 score (assessing psychological distress) as the mediated variable, and PCL-5 (assessing PTSD) as the dependent variable. Moreover, gender (reference group of males), teaching experience (reference group of 5 years and below), and school type (reference group of public school) were treated as confounding variables (Figure 1). The bootstrapping method was used to examine whether the mediation effects were significant in the tested model via 5000 bootstrap samples. Moreover, the 95% confidence internal (CI) of the bootstrap samples was used: when the upper limit CI (ULCI) and lower limit CI (LLCI) do not cover 0, the mediated effect is significant [41]. All the analyses were performed using the SPSS 20.0 (SPSS Inc.; Chicago, IL, USA) [42].

## 3. Results

The participants who agreed to participate in the study (N = 2603) completed all items of the online survey. The sample size is sufficient, as it is larger than the calculated size (i.e., 2377). Among participants, approximately one-quarter were males (28.4%), and almost all participants taught in public schools (99.5%). Slightly more than half of the participants had 10 or fewer years of teaching experience (56.2%). On average, the fear of COVID-19 score was 15.40 (out of 35; SD = 4.20), the nomophobia score was 69.14 (out of 140; SD = 28.84), the psychological distress score was 30.03 (out of 63; SD = 10.99), and the score for PTSD was 14.46 (out of 80; SD = 12.66). Using the cutoff of 31, the prevalence of PTSD was 12.3%, but 1% when using the cutoff score of 49 (see Table 1).

Table 2 presents the bivariate correlation coefficients between the studied variables. More specifically, the fear of COVID-19 score, nomophobia score, psychological distress score, and PTSD score were all moderately and significantly associated with each other (*r* = 0.26 to 0.58; all *p*-values < 0.001).

Hayes’ PROCESS Model 8 showed that the fear of COVID-19 score significantly explained the psychological distress score (coefficient = 5.72, SE = 0.81; *p* < 0.001) and PTSD (coefficient = 4.75, SE = 0.82; *p* < 0.001). The nomophobia score (coefficient = 0.09, SE = 0.02; *p* < 0.001) and DASS-21 (coefficient = 0.57, SE = 0.02; *p* < 0.001) significantly explained the PTSD score. Moreover, the results of moderated direct effect indicated that nomophobia moderated the positive association between fear of COVID-19 and PTSD (coefficient = 0.001, SE = 0.01; *p* = 0.047; Figure 1) whereas the moderated indirect effect of nomophobia on fear of COVID-19 to psychological distress is non-significant (coefficient = 0.002, SE = 0.01; *p* = 0.87). The indirect effect of fear of COVID-19 on PTSD via psychological distress was significant (coefficient = 3.32, SE = 0.33; bootstrapping lower limit of confidence interval at 95% = 2.71; bootstrapping upper limit of confidence interval at 95% = 4.00) (Table 3).

## 4. Discussion

To the best of the authors’ knowledge, the present study is the first to examine PTSD among teachers from k-12 schools during the COVID-19 pandemic. Considering the need to address the scarcity of research into PTSD among schoolteachers, the study shed light on the prevalence of PTSD resulting from COVID-19. The prevalence of COVID-19 related PTSD was 12.3%, using the cutoff score of 31, which decreased to 1% with a cutoff score of 49%. This is lower than that reported in a cross-sectional study that examined the prevalence of PTSD among teachers (24.4%) three months after the Lushan earthquake [43], and more than was found in a study of PTSD among Chinese college students (2.7%) one month after the outbreak of COVID-19 [21].

The present study’s findings demonstrated significant positive correlations between fear of COVID-19 and psychological distress. However, it should be kept in mind that the level of stress could be linked to uncertainty about the possibility of children becoming infected in schools. Although there are no similar studies among teachers, this relationship concurs with prior studies involving different populations [12]. For instance, in a study involving the general Turkish population, increased fear of COVID-19 was strongly associated with negative emotional states, including anxiety, depression, and stress [44]. Other previous studies have shown a similar pattern of significant positive relationship between fear of COVID-19 and psychological distress [44,45,46]. Researchers, along with medical and psychiatric bodies (e.g., World Health Organization and American Psychiatric Association), have acknowledged the influence of fear of COVID-19 on several aspects of teaching condition including burnout [47,48]. In the educational field, burnout syndrome among teachers occurs as prolonged stress can be caused by changes in their professional environment, as has been the case with the COVID-19 pandemic [49]. The consequences of the pandemic can cause changes in motivation, producing attrition and weakening the ability to regulate internal emotional responses [50]. Therefore, future studies are encouraged to examine the association between fear of COVID-19 and its potential influence on teaching burnout.

Additionally, the findings indicated significant positive relationships between fear of COVID-19 and PTSD. Prior studies have shown a consistent association between fear of COVID-19 and PTSD [21,24]. The present study advanced this knowledge by extending the evidence in the pandemic to a schoolteacher cohort. Many individuals may have feared the contagious nature of COVID-19 that they feared would unwittingly infect and consequently spread the disease to their family members. Therefore, psychological interventions that reduce the fear of the pandemic and instill emotional resilience among individuals exposed to the pandemic could help prevent PTSD or depressive symptoms.

In addition, the present study analyzed the association between fear of COVID-19 and PTSD and found that the association was mediated by psychological distress. The study’s mediation analysis indicated that teachers with a fear of COVID-19 were more likely to have psychological distress, which ultimately led to PTSD. The fear of COVID-19 can affect experiences of stigmatization and social exclusion of confirmed patients, survivors, their families, and others associated with the disease, which increases the risk of developing psychological distress [51]. Furthermore, uninfected individuals have reported that they are afraid of have contact with COVID-19-infected individuals [52]. While fear is considered helpful in motivating individuals to respond effectively to a given threat or negative stimuli, extreme and persistent fear may result in negative psychological reactions such as stress, depression, and anxiety [53]. Therefore, to address teachers’ emotional and mental health needs during the COVID-19 pandemic, existing instructional technology tools [54] or psychological intervention can be rapidly adapted to support trauma-informed educational practices.

Moreover, the present study indicated that nomophobia played a moderating role in the direct effect between fear of COVID-19 and PTSD. Based on the nomophobia score, nomophobia moderated the association of fear of COVID-19 on PTSD. A potential reason is that an individual with high levels of nomophobia and fear of COVID-19 may use a smartphone to search for coping methods to deal with the fear of COVID-19. For instance, a teacher has higher levels of nomophobia under the situation of COVID-19 as indicated by the fear of COVID-19, the teacher may be motivated to retain, protect, and build various resources to survive and maintain well-being. However, such protection may work only for a serious condition (e.g., PTSD) but not a mild condition (e.g., psychological distress) [55]. Thus, study findings explain why significant moderated effects on PTSD but not on psychological distress. The statement can be supported by the Demand-Control model and the Conservation of Resources (COR) theory [56]. Nomophobia may trigger an individual experiencing fear of COVID-19 to use a smartphone to seek online help, which should be further investigated.

The present study has some limitations. First, the use of cross-sectional design cannot provide strong evidence for causality. Therefore, future research should employ longitudinal designs to overcome this. Secondly, this study used the convenience sampling method and was unable to address response bias, which limits the representativeness of the present sample. In order to obtain more generalizable result, future studies are advised to use more nationally representative sample groups. Thirdly, reported PTSD use may be vulnerable to inherent bias due to self-report. As individuals with persistent PTSD recall the traumatic event, their recall may be biased by their judgement, and they selectively retrieve information that is consistent with these judgements [57]. Therefore, PTSD could be exaggerated compared to that expected which could lead to an overestimate of PTSD prevalence. However, conversely, the recruitment method (i.e., utilizing school principals to collect convenience samples) may have led to a lower level of PTSD than expected. More specifically, the principals who agreed to participate in the present study might have been confident in their teachers’ psychological resilience and coping skills during the COVID-19 outbreak period. Therefore, the present study might be biased by a sample comprised of participants with relatively good mental health and a lower likelihood of PTSD. Despite all these limitations, the PCL-5 (i.e., the measure assessing PTSD) used in the present study has high sensitivity and specificity.

## 5. Implications

The present study suggests that more should be done to lessen the risk of PTSD among k-12 schoolteachers. These findings suggest that educational authorities should allocate reasonable resources to identifying and helping k-12 schoolteachers who are at high risk of developing PTSD. There is a need for trauma-informed educational practices and adaptive formative assessment tools to support teachers’ mental health during the ongoing COVID-19 pandemic period. The k-12 school administrators need to support teachers’ mental health needs through trauma-informed teaching, which requires ensuring safety, establishing trustworthiness, maximizing choice, maximizing collaboration, and prioritizing empowerment [50].

## 6. Conclusions

The present cross-sectional study provided novel data concerning post-traumatic psychological distress related to COVID-19 in China, and suggests a need for more general psychological support, especially in educational settings. The study also found that fear of COVID-19 among teachers appears to result in PTSD via psychological distress, highlighting the moderating effect of nomophobia in this association. The study also provided the foundational evidence needed to help formulate psychological interventions to improve teachers’ mental health and psychological adaptability during the COVID-19 pandemic, as well as similar pandemics in the future.

## Figures and Tables

**Table 1:** Participant characteristics (N = 2603).

Variables	Mean (SD) or n (%)
Gender (male)	738 (28.4)
School type (public)	2591 (99.5)
Teaching experience in years	
(*i*) *5 years and below*	907 (34.8)
(*ii*) *6*–*10 years*	556 (21.4)
(*iii*) *11*–*15 years*	306 (11.8)
(*iv*) *16*–*20 years*	251 (9.6)
(*v*) *21 years and above*	583 (22.4)
Fear of COVID-19 Scale score	15.40 (4.20)
Nomophobia Questionnaire score	69.14 (28.84)
Depression, Anxiety, Stress Scale score	30.03 (10.99)
^1^ PTSD Checklist for DSM-5 score	14.46 (12.66)
Probable ^1^ PTSD (yes; using a cutoff score of 31 or above)	321 (12.3)
Probable ^1^ PTSD (yes; using cutoff score of 49 or above)	27 (1.0)

^1^ PTSD = post-traumatic stress disorder.

**Table 2:** Correlation matrix for studied variables.

	*r* (*p*-Value)						
	Gender	School Type	Teaching Experience	FCV-19S	NMPQ	DASS-21	PCL-5
Gender	-						
School Type	0.19 (<0.001)	-					
Teaching Experience	−0.35 (<0.001)	−0.18 (<0.001)	-				
FCV-19S	0.06 (0.002)	0.02 (0.31)	0.03 (0.21)	-			
NMPQ	0.09 (<0.001)	<0.001 (0.998)	0.01 (0.81)	0.34 (<0.001)	-		
DASS-21	−0.10 (<0.001)	−0.02 (0.29)	0.12 (<0.001)	0.37 (<0.001)	0.26 (<0.001)	-	
PCL-5	−0.09 (<0.001)	−0.08 (<0.001)	0.04 (0.053)	0.37 (<0.001)	0.28 (<0.001)	0.58 (<0.001)	-

FCV-19S = Fear of COVID-19 Scale; NMPQ = Nomophobia Questionnaire; DASS-21 = Depression, Anxiety, Stress Scale; and PCL-5 = PTSD Checklist for DSM-5.

**Table 3:** Results of Hayes’ PROCESS Model 8.

	Coefficient (SE)/LLCI, ULCI (*p*-value)	
	DASS-21 (R^2^ = 0.18)	PCL-5 (R^2^ = 0.38)
FCV-19S	5.72 (0.81)/4.12, 7.30 (<0.001)	4.75 (0.82)/3.14, 6.36 (*p* < 0.001)
NMPQ		0.09 (0.02)/0.04, 0.14 (*p* < 0.001)
DASS-21		0.57 (0.02)/0.53, 0.61 (*p* < 0.001)
Moderated Effect of NMPQ on FCV-19S to PCL-5		0.001 (0.01)/−0.04, −0.0002 (*p* = 0.047)
Moderated Effect of NMPQ on FCV-19S to DASS-21		0.002 (0.01)/−0.02, 0.02 (*p* = 0.87)
Indirect Effect Via DASS-21		3.32 (0.33)/2.71, 4.00 ^a^

Gender, teaching experience, and school type were controlled in the models. LLCI = lower limit of confidence interval at 95%; ULCI = upper limit of confidence interval at 95%; FCV-19S = Fear of COVID-19 Scale; NMPQ = Nomophobia Questionnaire; DASS-21 = Depression, Anxiety, Stress Scale; and PCL-5 = PTSD Checklist for DSM-5. ^a^ Using 5000 bootstrap samples for LLCI and ULCI; therefore, no *p*-value can be reported.

## Data Availability

The data presented in this study are available on request from the corresponding author. The data are not publicly available due to the restriction by the institutional review board.
